# How to turn up the heat on the cold immune microenvironment of metastatic prostate cancer

**DOI:** 10.1038/s41391-021-00340-5

**Published:** 2021-04-05

**Authors:** Jacob Stultz, Lawrence Fong

**Affiliations:** Division of Hematology/Oncology, Department of Medicine and Helen Diller Family Comprehensive Cancer Center, University of California San Francisco, San Francisco, CA USA

**Keywords:** Cancer therapy, Translational research

## Abstract

**Background:**

Advanced prostate cancer remains one of the most common and deadly cancers, despite advances in treatment options. Immunotherapy has provided little benefit to a majority of patients, largely due to the immunosuppressive tumor microenvironment that gives rise to inherently “cold tumors”. In this review, we discuss the immunopathology of the prostate tumor microenvironment, strategies for treating prostate cancer with immunotherapies, and a perspective on potential approaches to enhancing the efficacy of immunotherapies.

**Methods:**

Databases, including PubMed, Google Scholar, and Cochrane, were searched for articles relevant to the immunology of prostate cancer. We discuss the impact of different types of treatments on the immune system, and potential mechanisms through which prostate cancer evades the immune system.

**Results:**

The tumor microenvironment associated with prostate cancer is highly immunosuppressive due to (1) the function of regulatory T cells, tumor-associated macrophages, and myeloid-derived suppressor cells (MDSCs), (2) the cytokine milieu secreted by tumor stromal cells and fibroblasts, and (3) the production of adenosine via prostatic acid phosphatase. Both adenosine and tumor growth factor beta (TGF-beta) serve as potent immunosuppressive molecules that could also represent potential therapeutic targets. While there have been many immunotherapy trials in prostate cancer, the majority of these trials have targeted a single immunosuppressive mechanism resulting in limited clinical efficacy. Future approaches will require the integration of improved patient selection as well as use of combination therapies to address multiple mechanisms of resistance.

**Conclusion:**

Prostate cancer inherently gives rise to multiple immunosuppressive mechanisms that have been difficult to overcome with any one immunotherapeutic approach. Enhancing the clinical activity of immunotherapies will require strategic combinations of multiple therapies to address the emerging mechanisms of tumor immune resistance.

## Introduction

Prostate cancer exists along a spectrum from localized hormone-sensitive to metastatic castration-resistant. Treatment options for localized disease include radical prostatectomy, radiation therapy, and androgen deprivation therapy (ADT). While advanced prostate cancer is often treated initially with ADT, a majority of patients progress to metastatic castration-resistant prostate cancer (mCRPC) [1]. The use of currently approved therapies for advanced prostate cancer, such as androgen-receptor axis-targeted agents, taxane chemotherapy, radium-223, and sipuleucel-T, has led to a limited improvement in overall survival in patients with mCRPC, but patients ultimately progress. The ability of immunotherapies to induce durable clinical responses in solid tumors such as melanoma and renal cell cancer [2, 3] has yet to be fully realized in prostate cancer. The limited efficacy of checkpoint inhibition in treating prostate cancer has highlighted the complex immunosuppressive nature of prostate cancer and its associated tumor microenvironment (TME) [4].

**Fig. 1:**
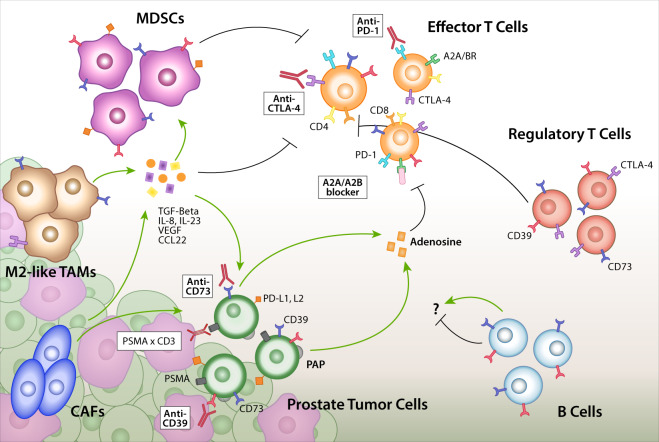
Interactions in the tumor microenvironment and effects of therapies. A2A/BR adenosine 2A/2B receptor, CCL22 C-C motif chemokine 22, CTLA-4 cytotoxic T lymphocyte-associated protein 4, IDO indoleamine 2,3-dioxygenase, IL-10 interleukin 10, MDSCs myeloid-derived suppressor cells, NOS nitric oxide synthase, PAP prostatic acid phosphatase, PD-1 programmed cell death protein 1, PD-L1 programmed death-ligand 1, PGE2 prostaglandin E2, TAMs tumor-associated macrophages, TGF-ß tumor growth factor beta, VEGF vascular endothelial growth factor. Shown here are some of the numerous interactions in the tumor microenvironment, both inhibitory and stimulatory, between different cell types, the cytokines and chemokines they produce, the local immune cells, and the many therapies used to treat prostate cancer, as detailed above.

The mechanisms through which prostate cancer maintains immune evasion and mediates immunosuppression continue to be elucidated. The results of these studies, discussed in more detail later, emphasize the network of cellular and molecular components that collectively enable prostate cancer to maintain a complex immunosuppressive nature and develop resistance to immunotherapies (Fig. 1). The cellular components include fibroblasts within the tumor stroma, regulatory T cells, tumor-associated macrophages (TAMs), and myeloid-derived suppressor cells (MDSCs). Collectively, cells within the TME express and secrete molecules that can mediate immunosuppression, and includes proteins such as programmed death-ligand 1 (PD-L1) with programmed cell death protein 1 (PD-1), tumor growth factor beta (TGF-beta), adenosine, IL-6, IL-8, IL-10, vascular endothelial growth factor (VEGF), and prostaglandin E2 [5].

In order to understand the strategies to best overcome resistance to immunotherapies in the setting of advanced prostate cancer, it is critical to understand (1) the cellular components of the TME responsible for immunosuppression, (2) the molecules that facilitate continued tumor growth, (3) the clinical experience with immunotherapies thus far, including likely methods of resistance, and (4) the current therapeutic strategies being studied to overcome barriers of response. This review examines these four aspects in order to outline the progress that has been made thus far in overcoming the immunosuppressive nature of prostate cancer and the areas in which the need for further investigation is most needed.

## Cellular components of the TME

### CAFs

Cancer-associated fibroblasts (CAFs) are the most abundant cell type within the TME [6]. They are known to play a significant role in the tumorigenesis and progression of prostate cancer, primarily through contact-dependent and paracrine signaling-mediated crosstalk between fibroblasts and surrounding prostate cancer cells [7–9].

Several in vitro and in vivo studies have highlighted the key signaling factors and the phenotypic changes that correlate with co-culturing fibroblasts with prostate cancer cells. As a result of the interaction, both cell populations secrete elevated levels of growth factors such as basic fibroblast growth factor, platelet-derived growth factor, and tumor necrosis factor alpha, and CAFs are induced to secrete matrix metalloproteases and VEGF. CAFs reciprocally induce prostate cancer cells to undergo a phenotypic shift, acquiring more stem cell-like properties and undergoing an epithelial–mesenchymal transition as evidenced by enhanced anchorage-independent growth and cell proliferation [7–9].

In vivo, CAF proliferation leads to the development of a fibrous stroma, which leads to local hypoxia and chronic inflammation via alterations in local vasculature [5]. In response, immunosuppressive cell populations such as regulatory T cells and MDSCs are recruited to the TME and further contribute to the maintenance of immune evasion and resistance to therapies seen with prostate cancer [5].

### Regulatory T cells

While fibroblasts are the most abundant cell type within the TME and are crucial for prostate cancer tumorigenesis and progression to mCRPC, regulatory T cells are regarded as one of the most important cell types responsible for the development and maintenance of an immunosuppressive TME [10, 11]. Prostate cancer tumors benefit from the activation and maintenance of regulatory T cells, as regulatory T cells are largely responsible for the attenuation of antitumor immune responses via contact- and cytokine-dependent suppression of antitumor cytotoxic T cells and tumor-infiltrating lymphocytes (TILs), natural killer cells, dendritic cells, and neutrophils [12].

In addition, while regulatory T cells are typically identified as CD4 + CD25 + FoxP3+ T cells, it has been shown that both CD4 + CD25+ and CD8 + FoxP3+ regulatory T-cell populations can be found within prostate cancer tumors. Both subsets of regulatory T cells have potent immunosuppressive capacities, including suppression of naïve T-cell proliferation in a contact-dependent manner [10]. Peripheral regulatory T-cell populations are present at levels similar to those observed in healthy individuals, yet they possess a more potent immunosuppressive ability and act via the same mechanisms seen within the TME [13].

### MDSCs

The immunosuppressive nature of regulatory T cells is bolstered by activation of MDSCs via TGF-beta, and VEGF. MDSCs represent a heterogeneous population of myeloid cells associated with chronic infection and malignancies that do not fully differentiate into granulocytes, dendritic cells, or macrophages [14–17]. They are recruited to the prostate cancer TME primarily through binding of C-X-C motif ligand 5 on prostate cancer cells with C-X-C motif receptor 2 on MDSCs, as well as through IL-8-mediated chemotaxis [18, 19]. The role of the latter has been more recently shown to be associated with poor prognosis and resistance to CPI therapy across cancer types, as well as within prostate cancer, highlighting MDSCs a potential contributor to the observed differences [20–23].

Functionally, MDSCs induce immunosuppression through upregulation of nitrous oxide synthase, arginase-1, and indoleamine 2,3-dioxygenase, which lead to the depletion of arginine and tryptophan from the TME. This effectively suppresses antitumor immune responses through T-cell cell-cycle arrest, decreased expression of T-cell receptor zeta chains, and downregulation of MHC class II molecules on antigen-presenting cells [24–27].

MDSCs and their impact on the TME were also shown to be critical for prostate cancer growth and development of castration resistance [18, 28]. A recent study utilizing IHC found that MDSCs were the most abundant tumor-infiltrating immune cell population within prostate cancer tumor samples. At the same time, inhibition of MDSC recruitment to and infiltration of prostate cancer tumors were sufficient to significantly delay prostate cancer progression in mouse models [18].

### TAMs

Macrophage recruitment and infiltration of prostate cancer tumors are mediated primarily through osteoclast secretion of CC chemokine ligand 2 (CCL2) in response to tumor-derived parathyroid hormone-related protein. This subsequently binds to CC chemokine receptor type 2 (CCR2) on monocytes within peritumor vasculature, acting as a potent chemoattractant [29]. This is especially relevant in the context of mCRPC, as metastatic nodules have been found to have an increased proportion of TAMs in contrast to primary tumors. In addition, in vitro and in vivo models have shown that inhibition of CCL2-mediated signaling via antibodies was sufficient to significantly hinder growth of bony metastases, and was associated with decreased proportions of TAMs within tumors [29, 30].

TAMs can be stratified into two distinct subpopulations, M1-like (CD163−CD206−) and M2-like (CD163+CD206+), based on their phenotypes and the environment in which they’re polarized. M1-like macrophages are typically activated by and involved with pro-inflammatory immune responses, while M2-like macrophages are associated with an anti-inflammatory phenotype [31]. While initial prognostic studies seemed to find little correlation between macrophages and patient outcomes, more recent evidence suggests that this was largely due to a lack of distinction between macrophage phenotypes. Differentiating M1-like from M2-like macrophages is crucial for understanding the impact of TAMs on the TME, since the different phenotypes can have starkly different effects on the antitumor immune response. This is evidenced by a study that examined the impact of TAMs on colorectal cancer, in which TAMs are preferentially polarized to M1-like, in contrast to prostate cancer, in which TAMs are mainly M2-like. Investigators found that M1-like TAMs were associated with increased tumor infiltration of type 1 helper and cytotoxic T cells, while M2-like TAMs were associated with a lack of effective T-cell function [32].

In studies that delineate these two subpopulations, there is consistent evidence that the abundance of M2-like TAMs is associated with tumor aggressiveness and poorer patient outcomes [31–33]. In addition, presence of M2-like TAMs within the TME was found to be an independent predictor of extracapsular extension in patients with prostate cancer, and the function of TAMs was shown to be independent of interactions with regulatory T cells within the TME [33].

## Mediators of immunosuppression

### CTLA-4

CTLA-4 is a prominent immune checkpoint present in prostate cancer. Under homeostatic conditions, CTLA-4 is expressed on T cells upon activation and constitutively on regulatory T cells. Expression of CTLA-4 is also upregulated on T cells that reach a state of exhaustion [34, 35]. CTLA-4 has a higher affinity for CD80 and CD86 than the co-stimulatory ligand CD28, and leads to suppression of T-cell activation through binding and effectively sequestering CD80 and CD86. Under physiologic conditions, this interaction takes place during the presentation of self-antigens to T cells. However, prostate cancer tumors benefit directly and indirectly through CTLA-4-mediated immunosuppression and a general anergic state [34].

Mechanistically, binding of CTLA-4 on T cells to CD80 or CD86 on antigen-presenting cells inhibits PI3K-mediated activation of Akt, interrupting the normal downstream signaling that occurs after the T-cell receptor (CD3) binds MHC molecules. Since PI3K signaling is necessary for T-cell survival, this effectively prevents CD3-dependent T-cell activation and effector function, including secretion of cytokines such as IL-2 and tumor necrosis factor alpha, without inducing T-cell apoptosis [34].

### PD-1/-L1

The programmed death receptor PD-1, along with its complementary ligands PD-L1 and PD-L2, act as inhibitory co-receptor-ligand pairs. Together, they serve to maintain balance between immune activation and immunosuppression, inducing T-cell anergy and mediating tolerance [36]. PD-1 is preferentially expressed on regulatory T cells, as well as natural killer cells and activated B cells. PD-L1, on the other hand, is expressed in a variety of tissues, including vascular endothelium, muscle, hepatocytes, placenta, pancreatic islet cells, epithelium, and mesenchymal stem cells. Within the immune system, PD-L1 is also expressed on B cells, T cells, dendritic cells, macrophages, and mast cells [37, 38].

Tumors, including prostate cancer, utilize this interaction through enhanced expression of PD-L1. This enables prostate tumors to induce the activation and maintenance of regulatory T cells within the TME, as well as suppress antitumor immune responses from effector T cells in a process termed immunoediting [39]. Expression of PD-L1 in tumor tissue has been significantly associated with poorer patient prognosis in the setting of prostate cancer. In one study, patients with prostate cancer and confirmed lymph node metastasis were stratified based on the expression of PD-L1, with high PD-L1 defined as ≥1% expression in tumor tissue. Patients with high levels of PD-L1 expression were found to experience significantly shorter metastasis-free survival (*p* = 0.008). Moreover, univariate analyses found that high PD-L1 expression was also significantly correlated with a higher risk of experiencing distant metastases (HR = 3.90) [40].

In another study that investigated correlations between PD-1, PD-L1, and clinical outcomes in patients with prostate cancer, it was found that both PD-1 and PD-L1 were significantly upregulated in malignant tissue when compared to benign tissue (PD-1–1.5% vs. 7.7%, *p* = 0.003; PD-L1–0.5% vs. 13.2%, *p* < 0.001). Akin to the aforementioned study, ≥1% PD-L1 expression in prostate tumors was associated with the presence of lymph node metastases, albeit the difference was nonsignificant (10.1% vs. 27.3%, *p* = 0.086). Interestingly, there was also significantly higher expression of PD-L1 in prostate tumors with higher pathological tumor (pT) staging. In patients with tumors classified as pT2/3a, ≥1% PD-L1 expression was observed in 11.4% of samples vs. 31.6% of samples in patients with pT3b/4 tumors (*p* = 0.013) [41]. Collectively, these studies highlight the prognostic relevance of PD-L1 expression in prostate cancer, suggesting a connection between higher PD-L1 levels and increased capacity for tumors to evade immune responses and progress to systemic disease.

### TGF-beta

TGF-beta is a pleotropic cytokine, mediating a range of functions. Under homeostatic conditions, TGF-beta can act as a bifunctional regulator of cell growth, including serving as a tumor suppressor molecule and potent regulator of the cell cycle. This is accomplished by preventing tumorigenic growth through maintained phosphorylation of the retinoblastoma protein, induction of p15 and p21 transcription, and inhibition of c-Myc-induced growth signaling [42]. However, in the context of tumorigenesis, TGF-beta carries the capacity to serve as a potent promoter of tumorigenesis and as a mediator for the recruitment of immunosuppressive cell populations. TGF-beta is overproduced by most tumors, including by prostate cancer cells, with contributions from tumor-associated cell populations such as CAFs and TAMs. It has been shown that TGF-beta can independently induce transformation of CAFs, and in the presence of epithelial growth factor, has the potential to induce anchorage-independent growth of other fibroblast cell lines [43]. CAFs, in response to TGF-beta, produce mitogenic signals that promote prostate cancer cell survival and proliferation. In addition to stimulating CAF-mediated fibrosis and promoting tumor progression, TGF-beta has also been shown to be an important signaling molecule in angiogenesis, metastasis, and immunosuppression [44, 45].

### Adenosine

Adenosine acts as an anti-inflammatory mediator and is activated in order to protect healthy tissue from inflammation-related damage. Nearly two decades ago, Sitkovsky et al. highlighted the various immunomodulatory effects of adenosine, primarily through activation of the adenosine A2 receptor (A2AR), and subsequently discovered that activation of the A2AR on antitumor T cells led to the inhibition of effector responses and contributed to tumor immune evasion [46, 47].

The adenosine signaling pathway could be especially important in prostate cancer, as adenosine is generated by prostatic acid phosphatase (PAP), and activation of the adenosine signaling pathway leads to attenuation of the impact of TILs, generating an immunosuppressive TME [48–50]. Adenosine has been evidenced to contribute to several other immunosuppressive and pro-tumorigenic factors aside from modulating the impact of CD8+ TILs. It also plays a role in limiting the functionality of dendritic cells, in supporting both tumor-promoting and angiogenic fibroblasts, and in increasing regulatory T cells and MDSCs [51–54].

One study highlighted the importance of the CD73-adenosinerigic pathway in prostate cancer immunosuppression. Comparisons of tumor vs. adjacent normal tissue stratified by CD73 expression revealed a significant correlation between CD73 levels and duration of biochemical recurrence-free survival: expression of CD73 above the cohort median in normal tumor-adjacent epithelium was shown to independently predict shorter biochemical recurrence-free survival (HR = 2.753, 95% CI = 1.483–5.109; *p* = 0.001). In addition, high densities of CD8+ lymphocytes within tumor tissue were correlated with shorter biochemical recurrence-free survival in the setting of elevated CD73 levels within normal adjacent epithelium [55]. This study further suggests that CD73 activity, including adenosine production, leads to an immunosuppressive impact on the TME.

## Clinical trials of immunotherapies and mechanisms of resistance

### Sipuleucel-T

Sipuleucel-T was the first FDA-approved immunotherapy for the treatment of asymptomatic or minimally symptomatic mCRPC, and thus understanding its impact on the immune system is critical in understanding how it can best be utilized with other therapies [56]. Patients undergo plasmapheresis, and their leukocytes are cultured with PA2024, a fusion protein consisting of PAP connected to granulocyte-macrophage colony-stimulating factor [57]. Consequently, this process leads to a statistically significant increase in antigen-presenting cell stimulation, with the largest response observed in patients with earlier stages of prostate cancer [58]. Thus, sipuleucel-T acts as an autologous cellular immunotherapy that leads to the induction of a PAP-targeted antitumor immune response, affecting both the TME and peripheral immune system [57].

In the periphery, reintroduction of the expanded and stimulated antigen-presenting cells causes an increase in systemic activation of cytotoxic T lymphocytes. At the same time, it increases trafficking of both T and B cells to tumor margins, as seen in a study carried out in men with localized prostate cancer that received neoadjuvant sipuleucel-T [59]. Rather than expanding preexisting T cells that are already present within the tumor, sipuleucel-T recruits new T cells to the tumor site, which is represented by a systemic reduction in T-cell receptor diversity alongside a simultaneous increase in T-cell receptor diversity within the TME [60].

Not only does treatment with sipuleucel-T leads to increased activity of T cells against PAP, it also causes an increased T-cell response to other tumor-associated antigens. After treatment, the resulting lysis of tumor cells gives rise to the release of tumor-specific antigens (TSAs), which further induce an antitumor immune response [57, 61]. This secondary immune response typically begins 2 weeks after treatment, and targets several key proteins for which expression is known to be upregulated in prostate cancer cells, such as prostate-specific antigen (PSA) and KLK2 [57, 62]. In addition, sipuleucel-T generates a robust immune response that primes the immune system, after which subsequent infusions further boost the response in an anamnestic manner. This has been shown through studies of men with mCRPC that received additional infusions of sipuleucel-T as their disease progressed, which led to increased amounts of antigen-presenting cell activation [63–65].

While initial clinical trials collectively showed a significant improvement in overall survival among patients that received sipuleucel-T, the benefit was modestly greater at most in comparison to other treatments approved for use in mCRPC. The clinical trials that led to the approval of sipuleucel-T were difficult to interpret due to the lack of significant change in time to disease progression despite improved overall survival, as well as discrepancies in baseline patient characteristics between treatment arms and other logistic issues [66].

### Anti-CTLA-4

With continued identification of targetable immune checkpoints and development of CPIs, such as an anti-CTLA-4 antibody, further clinical trials were conducted that evaluated them in the setting of prostate cancer (Table 1). Originally, adjuvant anti-CTLA-4 therapy following surgery was shown to be an effective therapeutic approach for targeting residual tumor cells in studies of transgenic mice [67]. Therapy with anti-CTLA-4 monoclonal antibodies has been investigated in both phase I/II trials, as well as two notable phase III trials in the setting of advanced prostate cancer—one in chemotherapy-naïve patients and the other in patients that had disease progression while on docetaxel [68, 69].

**Table 1:** Immunotherapy clinical trials in prostate cancer.

Patient population	Study drugs	Combination type	Phase	Status	NCT
Anti-CTLA-4					
mCRPC	Apalutamide + abiraterone; apalutamide + abiraterone + ipilimumab; apalutamide + abiraterone + cabazitaxel + carboplatin	Anti-androgen, chemotherapy	II	Active, not recruiting	NCT02703623
mCRPC	Abiraterone + ipilimumab	Anti-androgen	I/II	Active, not recruiting	NCT01688492
HSPC	Ipilimumab + degarelix + radical prostatectomy				
mCRPC	Ipilimumab + valemetostat (EZH2 inhibitor)	Epigenetic modifier	I	Recruiting	NCT04388852
Anti-PD-1/PD-L1					
mCRPC	Pembrolizumab ± enzalutamide	Anti-androgen	II	Active, not recruiting	NCT02787005i
mCRPC	Enzalutamide ± pembrolizumab	Anti-androgen	III	Recruiting	NCT03834493
mCRPC	Enzalutamide + pembrolizumab	Anti-androgen	II	Active, not recruiting	NCT02312557
mCRPC	Avelumab + abiraterone; avelumab + enzalutamide	Anti-androgen	II	Active, not recruiting	NCT03770455
mCRPC, mHSPC	Nivolumab + rucaparib; nivolumab + docetaxel; nivolumab + enzalutamide	Anti-androgen PARP inhibitor, chemotherapy	II	Active, not recruiting	NCT03338790
mCRPC	Pembrolizumab + olaparib; pembrolizumab + docetaxel; pembrolizumab + enzalutamide; pembrolizumab + abiraterone; pembrolizumab + lenvatinib (VEGF inhibitor); MK7684 (pembrolizumab + vibostolimab [anti-TIGIT monoclonal antibody])	Anti-androgen, chemotherapy, PARP inhibitor	I	Recruiting	NCT02861573
mCRPC	Pembrolizumab + ^177^Lu-PMSA	Radiation	I/II	Active, not recruiting	NCT03658447
mCRPC	Radium-223 ± pembrolizumab	Radiation	II	Active, not recruiting	NCT03093428
mCRPC	Docetaxel ± pembrolizumab	Chemotherapy	III	Recruiting	NCT03834506
mCRPC	Pembrolizumab + docetaxel or cabazitaxel	Chemotherapy	II	Recruiting	NCT03248570
mCRPC	Pembrolizumab + guadecitabine (hypo-methylating agent)	Chemotherapy	I	Active, not recruiting	NCT02998567
Locally advanced, mCRPC	Pembrolizumab + etoposide + carboplatin/cisplatin; pembrolizumab + etoposide + carboplatin + docetaxel	Chemotherapy	I	Recruiting	NCT03582475
mCRPC	Rucaparib; nivolumab; rucaparib + nivolumab	PARP inhibitor	I/II	Active, not recruiting	NCT03572478
mCRPC	Pembrolizumab + olaparib; enzalutamide or abiraterone (opposite from previously received)	PARP inhibitor, anti-androgen	III	Recruiting	NCT03834519
Locally advanced, recurrent, mCRPC	Durvalumab + olaparib + cediranib; durvalumab + olaparib; durvalumab + cediranib	PARP inhibitor, antiangiogenic	I/II	Recruiting	NCT02484404
Locally advanced, mCRPC	Avelumab + talazoparib	PARP inhibitor	II	Active, not recruiting	NCT03330405
mCRPC	Cabozantinib + atezolizumab; abiraterone or enzalutamide	Antiangiogenic, anti-androgen	III	Recruiting	NCT04446117
Locally advanced, HSPC, CRPC, metastatic	XmAb22841 (anti-LAG3xCTLA-4 BSAb) ± pembrolizumab		I	Recruiting	NCT03849469
mCRPC	Pembrolizumab + HER2Bi-armed activated T cells	Adoptive cell therapy	II	Recruiting	NCT03406858
CRPC	Avelumab + PF-04518600 (anti-OX40 antibody); avelumab + utomilumab (4-1BB antibody); avelumab + PF-04518600 + utomilumab; RT + avelumab + PF-04518600 (anti-OX40 antibody); RT + avelumab + utomilumab (4-1BB antibody); RT + avelumab + PF-04518600 + utomilumab	Radiation, immune checkpoint inhibitors	I/II	Recruiting	NCT03217747
Localized	Atezolizumab ± tocilizumab (IL-6 antagonist)	Cytokine-targeting therapy	II	Recruiting	NCT03821246
mCRPC	Pembrolizumab + navarixin (CXCR1/2 antagonist)	Cytokine-targeting therapy	II	Active, not recruiting	NCT03473925
Localized, HSPC	Atezolizumab + PROSTVAC V/F + MVA-BN-Brachyury	Antitumor vaccine	II	Recruiting	NCT04020094
mCRPC	Nivolumab + stereotactic body radiation therapy + CDX-301 (recombinant FMS-like tyrosine kinase-3 ligand) + poly-ICLC (TLR3 agonist); nivolumab + NKTR-214 (IL-2 agonist); nivolumab + CDX-301 + INO-5151 (PSA and PMSA-targeting antigens with vector expressing IL-12)	Cytokine-targeting therapy, radiation	I	Recruiting	NCT03835533
Anti-CTLA-4 + anti-PD-1/PD-L1					
mCRPC	Nivolumab + ipilimumab + cyclophosphamide + cryosurgical freezing	Chemotherapy	II	Recruiting	NCT04090775
mCRPC	Durvalumab + tremelimumab + metronomic vinorelbine	Chemotherapy	I/II	Recruiting	NCT03518606
mCRPC	PROSTVAC V/F + nivolumab + ipilimumab + neoantigen DNA vaccine administered via electroporation	Antitumor vaccine	I	Recruiting	NCT03532217
mCRPC	Durvalumab + tremelimumab		II	Active, not yet recruiting	NCT03204812
mCRPC	Nivolumab + ipilimumab; ipilimumab; cabazitaxel	Chemotherapy	II	Recruiting	NCT02985957
Metastatic HSPC	Docetaxel + ADT; nivolumab + docetaxel + ADT; ipilimumab + nivolumab + docetaxel + ADT	Chemotherapy, anti-androgen	II/III	Active, not recruiting	NCT03879122
Stage IV prostate cancer	Ipilimumab + nivolumab + cabozantinib	Antiangiogenic	II	Recruiting	NCT03866382
Adenosine pathway inhibitors					
mCRPC	CPI-006 (anti-CD73); CPI-006 + pembrolizumab; CPI-006 + ciforadenant	Anti-CD73	I	Recruiting	NCT03454451
mCRPC	Ciforadenant ± atezolizumab	Anti-CD73	I	Recruiting	NCT02655822
Locally advanced, mCRPC	AZD4635 (A2AR antagonist) + oleclumab (anti-CD73); AZD4635 + durvalumab	Anti-CD73	II	Recruiting	NCT04089553
Locally advanced, mCRPC	AZD4635 + durvalumab; AZD4635 + durvalumab + oleclumab; AZD4635 + docetaxel; AZD4635 + enzalutamide; AZD4635 + abiraterone	Anti-CD73, chemotherapy, anti-androgen	I	Active, not recruiting	NCT02740985
mCRPC	Etrumadenant (A2A/BR antagonist) + zimberelimab (anti-PD-1) + enzalutamide; etrumadenant + zimberelimab + AB680 (CD73 inhibitor); etrumadenant + zimberelimab + docetaxel; etrumadenant + AB680; etrumadenant + zimberelimab	Anti-androgen, chemotherapy, anti-CD73	I/II	Recruiting	NCT04381832
Bispecific antibodies					
mCRPC	AMG 160 (anti-PMSAxCD3) ± pembrolizumab	Bispecific antibody	I	Recruiting	NCT03792841
mCRPC	AMG 160 + enzalutamide; AMG 160 + abiraterone; AMG 160 + AMG 404 (PD-1 inhibitor); AMG 404	Bispecific antibody, anti-androgen	I	Not yet recruiting	NCT04631601
mCRPC	Cemiplimab + REGN5678 (anti-PSMAxCD28)	Bispecific antibody	I/II	Recruiting	NCT03972657
Neuroendocrine	AMG 757 (anti-delta-like ligand 3xCD3)	Bispecific antibody	I	Not yet recruiting	NCT04702737
mCRPC	AMG 509 (anti-STEAP1xCD3)	Bispecific antibody	I	Recruiting	NCT04221542
mCRPC	HPN-424 (anti-PSMAxCD3)	Bispecific antibody	I/II	Recruiting	NCT03577028
mCRPC	JNJ-70218902	Bispecific antibody	I	Recruiting	NCT04397276
CART T cells					
mCRPC	PSMA-targeted CART/TGFβR dominant negative		I	Recruiting	NCT03089203
mCRPC	PSCA-targeted CART		I	Recruiting	NCT03873805

In the chemotherapy-naïve setting, the efficacy and safety of ipilimumab were studied in patients with mCRPC, excluding those with visceral metastases, that had not received any prior chemotherapy by randomizing them to receive either ipilimumab or placebo (with standard-of-care therapy). While the difference between treatment arms in regard to overall survival was nonsignificant (28.7 vs. 29.7 months, respectively; HR 1.1; 95% CI 0.88–1.39; *p* = 0.3667), it was found that treatment with ipilimumab compared to standard-of-care therapy was still associated with a significant improvement in progression-free survival (PFS) (5.6 vs. 3.8 months, HR 0.67, 95.87% CI 0.55–0.81) as well as in PSA response (23% vs. 8%). Thus, despite evidence of ipilimumab leading to a survival benefit in an unselected patient population, there was still evidence that ipilimumab generated an enhanced antitumor immune response.

Modestly positive phase I/II clinical trial results, in conjunction with growing excitement around CPIs due to their success in treating melanoma, led to a large-scale phase III clinical trial that also investigated ipilimumab for the treatment of mCRPC. In this trial, patients with mCRPC and at least one bone metastasis that had clinical disease progression despite treatment with docetaxel were randomized to receive ipilimumab or standard-of-care therapies. Due to studies at the time that suggested an amplified benefit from ipilimumab when used alongside radiation therapy, patients in this trial received one dose of radiation for one to five bone fields followed by randomization to either ipilimumab or placebo [70, 71]. Similar to the other phase III trial of ipilimumab, median overall survival for patients in this study resulted in a nonsignificant difference, with patients that were treated with ipilimumab realizing an 11.2-month median overall survival compared to 10 months for patients that received placebo (HR 0.85; 95% CI 0.72–1.00; *p* = 0.053). However, a piecewise model of the hazard ratio demonstrated an initial detrimental impact of ipilimumab from 0 to 5 months (HR 1.46, 95% CI 1.10–1.95), followed by a benefit from 5 to 12 months (HR 0.65, 95% CI 0.50–0.85) and beyond 12 months (HR 0.60, 95% CI 0.43–0.86) [68]. Exploratory analyses after trial completion led to the discovery that patients in which ipilimumab had the highest potential to benefit could be identified using several prognostic features: hemoglobin concentration ≥11.0 g/dL, alkaline phosphatase concentrations ≤1.5 times the upper limit of normal, and absence of visceral metastases. The absence of visceral metastases was the largest factor in predicting prognosis, and the only to independently interact with treatment. Post hoc evaluation of patients within the trial that fit these criteria showed that this subgroup had a significant improvement in overall survival (HR 0.623, 95% CI 0.45–0.86; *p* = 0.0038). In addition, a Cox covariate analysis revealed that overall survival was also impacted by the number of bone regions involved by bony metastases, with patients that had involvement ≥2 bone regions experiencing worse overall survival [68].

Together, the two major phase III clinical trials that investigated ipilimumab demonstrated that its use in the setting of unselected patients with mCRPC is met with inherent resistance. Since patients within the chemotherapy-naïve trial did not have visceral metastases, this trial effectively bolstered the proportion of patients that fit the potentially favorable prognostic features identified in the chemotherapy-resistant phase III trial. This difference offers a potential connection between the development of visceral metastases and increased resistance to anti-CTLA-4 CPI therapy. This trend was also present in the chemotherapy-naïve setting amongst patients that had metastases to more than one bone region. Development of metastases is largely facilitated by leukocytes and other immunomodulatory cell populations, such as fibroblasts and M2-like TAMs, and thus the presence of visceral disease and/or more extensive bony disease could represent a distinct immunophenotype or TME that imposes a higher barrier to antitumor immune responses.

While treatment with anti-CTLA-4 therapy showed clinical activity in patients with mCRPC, preclinical studies have highlighted the potential for general suboptimal generation of antitumor immunoreactivity, postulating several potential responsible mechanisms. For example, inhibition of CTLA-4 has not been shown to lead to any significant reduction in regulatory T cells. Several studies demonstrated that, despite significant changes in CD4+ and CD8+ effector T-cell populations, CTLA-4 blockade did not lead to an apparent reduction in regulatory T-cell population size. This was associated with limited efficacy of anti-CTLA-4 monotherapy except in a small subset of patients with prostate cancer [72–75].

At the same time, treatment with ipilimumab has been shown to upregulate expression of PD-L1 and V-domain immnunoglobulin suppressor of T-cell activation in CD4+ T cells, CD8+ T cells, and CD68+ macrophages within prostate cancer tissue samples compared to untreated samples [76]. Thus, prostate cancer tumors can potentially maintain sufficient immunosuppression through the immune changes associated with development of metastatic disease, enhanced recruitment and survival of immunosuppressive cells in the TME, and/or elevated expression of inhibitory immune checkpoint molecules aside from CTLA-4.

### Anti-PD-1/PD-L1 therapy

Over the last two decades, the excitement around immune CPIs has grown tremendously, especially around prostate cancer after sipuleucel-T received FDA approval [77]. This excitement, in conjunction with a persistent need for better therapies, has led to an increase in the number of clinical trials assessing the efficacy of immunotherapies beyond sipuleucel-T (Table 1). Currently, the IMBassador250 trial is the only phase III clinical trial that has investigated the use of anti-PD-1/PD-L1 antibodies in the setting of mCRPC. IMBassador250 evaluated the safety and efficacy of atezolizumab, an anti-PD-L1 antibody, with enzalutamide, an androgen-receptor antagonist, compared to enzalutamide alone in 759 patients. The trial was terminated early after finding that there was no observed difference in regard to the primary endpoint, overall survival, nor any of the secondary endpoints. This continued to be the trend between treatment groups and within predefined subgroups of patients [78].

While secondary and exploratory analyses are still ongoing for IMBassador250, similar phase I/II clinical trials that involve anti-PD-1/PD-L1 antibodies, including the trials that led up to IMBassador250, serve to highlight mechanisms of prostate cancer resistance to anti-PD-1/PD-L1 CPIs. The first trial of anti-PD-1 antibodies as monotherapy for mCRPC resulted in a lack of evidence of radiographic response in any of the 17 patients [79]. This lack of response, alongside preclinical studies that highlighted the negative prognosis associated with increased tumor PD-L1 expression, led to continued clinical trials of anti-PD-1/PD-L1 antibodies in more selective patient populations.

In the phase II clinical trial that preceded IMBassador250, patients with mCRPC and evidence of progression on enzalutamide were treated with pembrolizumab. Of the initial ten patients, three had a ≥50% reduction in PSA, and of those three, two had evidence of radiographic response [80]. Subsequent data from a larger cohort of patients from the same trial showed similar results; five patients (18%) had a ≥50% reduction in PSA, and of the 24 patients with measurable disease, six (25%) had evidence of radiographic response [81].

Exploratory analyses found that when contrasting groups, responders were found to have significantly higher baseline expression levels of granzyme B within CD8+ effector memory cells (*p* = 0.0081), and a similar nonsignificant trend for baseline perforin expression (*p* = 0.0695). This trend did not persistent when groups were compared with respect to proportion of MDSCs [81]. Altogether, these studies emphasize the immunosuppressive nature of prostate cancer that acts to impose a high barrier to developing effective antitumor immunogenicity. Of the five patients that did respond, one was found to have microsatellite instability with a tenfold increase in mutation frequency in tumor tissue. The other responders had evidence of a baseline antitumor cytotoxic T-cell population that was present prior to initiation of treatment that did not exist in non-responders [81].

The KEYNOTE-028 and KEYNOTE-199 trials similarly investigated the efficacy and safety of pembrolizumab in settings outside of anti-androgen therapies. KEYNOTE-028 evaluated pembrolizumab in patients with mCRPC found to be PD-L1+. KEYNOTE-028 was a phase Ib clinical trial that enrolled 35 patients with advanced solid tumors, as well as RECIST-measurable disease and ≥1% PD-L1 expression in tumor or stromal cells. Of these 35 patients, there was found to be a response in six (objective response rate = 17.4%, 95% CI = 5.0–28.8%) and confirmed radiographic response in seven (20%) [82]. These findings were unable to be replicated in KEYNOTE-199, a similarly designed phase II clinical trial that assessed the efficacy of pembrolizumab in patients that had prior exposure to docetaxel. Stratifying patients based on PD-L1 led to only a minimal difference in objective response rates (5% in PD-L1+ vs. 3% in PD-L1−). Subsequent exploratory analysis within the KEYNOTE-199 trial highlighted a possible association between alterations in DNA damage repair (DDR) genes such as *BRCA2* and *ATM*, and response to pembrolizumab. Patients with a mutation in either *BRCA2* or *ATM* had an objective response rate of 11% compared to 3% in patients without mutations in any of the 14 sequenced DDR genes [83].

Thus, these data suggest that prostate cancer resistance to PD-1/PD-L1-targeted CPI is primarily due to immunosuppression of antitumor cytotoxic T cells and can be overcome by increasing the capacity for the immune system to mount an antitumor response, either through increased tumor immunogenicity or increased abundance of tumor-reactive T cells. In addition, the lack of difference in MDSC population sizes between responders and non-responders points toward other mechanisms of maintained immunosuppression.

Further investigations into mechanisms of resistance found that there was a common tumor phenotype associated with lack of response. In one study, a subset of patients with bladder cancer that did not respond to anti-PD-1/PD-L1 therapy was found to have tumors with similar characteristics. This phenotype, which parallels a phenotype observed in other patients with urothelial cancers, involves exclusion of CD8+ TILs from the TME and high expression levels of TGF-beta in fibroblasts. This same study created a tumor-harboring mouse model with these characteristics, and elucidated that therapeutic inhibition of TGF-beta in conjunction with anti-PD-L1 antibodies led to reversal of the observed phenotype: reduction of TGF-beta expression in tumor stromal cells, increased tumor infiltration by CD8+ T cells, and enhanced generation of antitumor immune responses [84]. This study further bolstered evidence that response to anti-PD-1/PD-L1 therapy is effectively inhibited without additional tumor characteristics or therapies that act to increase tumor immunogenicity.

### Combination anti-CTLA-4 and anti-PD-1/PD-L1

While there have been many trials assessing the efficacy and safety of CPI monotherapy in the setting of prostate cancer, the overall observed benefit has been limited, as described earlier. Moreover, recent preclinical data revealed that treatment with combination anti-PD-1 and anti-CTLA-4 immunotherapy increased antitumor immune activity in mice with colon adenocarcinoma [85]. Together, the prior evidence of limited efficacy of CPI monotherapy in treating prostate cancer and the promising preclinical data for dual CPI therapy have led to multiple clinical trials involving dual anti-CTLA-4 and anti-PD-1/PD-L1 CPI therapy for the treatment of mCRPC. While there has yet to be any completed phase III clinical trials, the phase II clinical trials involving the use of both anti-CTLA-4 and anti-PD-1/PD-L1 highlight the subverted mechanisms of resistance, areas of persistent treatment resistance.

The CheckMate 650 trial was a phase II clinical trial evaluating the combination of ipilimumab and nivolumab (anti-CTLA-4 and anti-PD-1 antibodies, respectively). Patients with mCRPC were enrolled and stratified into two cohorts based on whether or not they had previously been treated with chemotherapy. In the 30 patients with measurable disease that had previously received chemotherapy, the objective response rate was 10% (95% CI = 2–27%). Comparatively, among the 23 patients with measurable disease and without previous chemotherapy exposure, there was a 25% objective response rate (95% CI = 10–48%). Analyses of patients with ≥1% PD-L1 expression found an increased objective response rate of 33.3% in patients that had previously received chemotherapy, and of 40% in patients that had not received prior chemotherapy. A broader, exploratory analysis of all the patients highlighted the importance of tumor mutational burden (TMB) and effective response to CPI therapies. In patients with a TMB above the median, the objective response rate was found to be 56.3%; in patients with alterations in DDR genes, the objective response rate was found to be 40% [86]. While there was evidence of significant clinical activity and comparatively more successful responses to treatment compared to clinical trials of CPI monotherapy, these results came at the cost of increased toxicity. Nearly all patients that received treatment experienced a treatment-related adverse event. Grade 3–4 treatment-related adverse events occurred in 42.2% of the chemotherapy-naïve cohort and in 53.3% of the chemotherapy-resistant cohort, and treatment-related deaths occurred in two patients from each cohort. These toxicities collectively led to treatment discontinuation in about half of patients from each cohort (51.1% and 44.4%, respectively). Thus, treatment-associated toxicities of the combination of ipilimumab and nivolumab at these doses represent a substantial barrier to further clinical development.

The CheckMate 650 trial showed that the combined use of anti-PD-1 and anti-CTLA-4 immunotherapies had clinical activity in patients with mCRPC, despite the associated toxicities. Factors that correlated with treatment-related benefit were collectively seen in the aforementioned clinical trials of anti-CTLA-4 or anti-PD-1/PD-L1 monotherapy, such as elevated expression of PD-L1 in tumor tissue, use in the chemotherapy-naïve setting, and increased TMB.

In a subsequent phase II clinical trial, 15 patients with mCRPC that were confirmed to express androgen-receptor splice variant 7 were treated with nivolumab plus ipilimumab. Preliminary data suggested an association between the expression of androgen-receptor splice variant 7 and the presence of dysfunctional DDR mechanisms. Thus, the trial attempted to enrich response to ipilimumab with nivolumab through the selection of patients more likely to have tumors with a higher TMB. Of the 15 patients that were enrolled, six were found to have mutations in DDR genes via targeted next-generation sequencing. Overall, the trial found that patients experienced an objective response rate of 25% in those with measurable disease (2/8), a PFS of 3.7 months (95% CI = 2.8–7.5 months), and an overall survival of 8.2 months (95% CI = 5.5–10.5 months). When stratified by functional status of DDR mechanisms, patients generally had a better prognosis with treatment if they were found to have aberrant DDR genes. However, most differences were nonsignificant with the exception of PSA-PFS and clinical/radiographic PFS (PSA-PFS: HR = 0.19, *p* < 0.01; PFS: HR = 0.31, *p* = 0.01) [87].

While this trial attempted to bolster response rates through patient selection, the observed differences were not substantial enough to reach statistical significance when contrasting patients with functional DDR genes against patients with aberrant DDR genes. The lack of a difference between sample groups could indicate mechanisms of resistance that negate the increased immunogenicity, such as increased expression of antiapoptotic pathways in tumor cells or dysfunctional antigen-presenting cells. At the same time, since androgen-receptor splice variant 7 is associated with poorer prognosis, it is likely that its presence leads to changes in tumor biology in a way that could impact the TME and/or response to CPI therapy [88].

Potential molecular mechanisms that could explain patient resistance to dual anti-PD-1 and anti-CTLA-4 CPI therapy include increased systemic levels of immunosuppressive cytokines, such as TGF-beta [23]. Recently, Jiao et al. demonstrated that TGF-beta serves a role in facilitating resistance to CPI therapy amidst bone metastases in the setting of mCRPC via inhibition of helper T-cell polarization to the Th1 phenotype. Patients that experienced a suboptimal response to CPI therapy were found to have bone marrow with increased Th17 and decreased Th1 helper T cells in contrast to those that responded to CPI therapy, suggesting that TGF-beta could contribute to attenuating antitumor immune responses via dampening of Th1 helper T cells.

In further experiments using mouse models of subcutaneous and osseous prostate cancer metastases, presence of bony metastases promoted TGF-beta production through induction of osteoclasts. While both models showed increased CD4+ TILs, mice with bony metastases experienced little benefit from CPI and were found to have increased levels of Th17 helper T cells. This was offset when CPI was co-administered with a TGF-beta inhibitor, allowing polarization of T cells to the Th1 phenotype and enhancing antitumor immune responses [89]. An analogous finding was discussed above in a study of patients with bladder cancer treated with anti-PD-1/PD-L1 CPI therapy.

Alternatively, resistance to dual anti-PD-1 and anti-CTLA-4 therapy could be derived from increased systemic levels of IL-8, which plays a crucial part in sustaining inflammation and recruiting cells such as MDSCs to the TME. Recent studies of patients from large-scale clinical trials have demonstrated that elevated IL-8 correlates with a poorer prognosis across cancer types as well as in prostate cancer specifically, for both anti-CTLA-4 and anti-PD-1/PD-L1 CPI therapies. IL-8 was also associated with diminished TILs, increased recruitment of immunosuppressive myeloid cells to the TME, and disrupted antigen-presenting cell function [20, 22, 23].

None of the potential mechanisms of resistance discussed are mutually exclusive, and thus additional, more granular data are needed in order to accurately differentiate the degree to which tumors employ each of these.

## Clinical strategies to enhance immune responses

Response to CPI therapy necessitates the generation of sufficient antitumor effector T cells, the proper function of antitumor cytotoxic T cells, and the formation of antitumor effector memory T cells. In addition, each stage relies on a series of processes: generation of tumor-specific cytotoxic T cells involves antigen-presenting cell recognition of tumor-associated neoantigens, internal processing of antigens, and antigen presentation. Thus, resistance to CPI therapy can result from the disruption of one or more of these three stages [90, 91].

In the first step of generating an optimal antitumor immune response, resistance can develop due to a lack of tumor-associated antigens available to antigen-presenting cells, immune cell inability to access the antigens, and dysfunctional antigen presentation. Prostate cancer and its associated TME can also disrupt antitumor T-cell function via immunosuppression from inhibitory checkpoint molecules like PD-L1, altered levels of molecules involved in metabolic and inflammatory pathways, and impaired tumor cell apoptosis. Improper or absent development of effector memory T cells can also lead to resistance, and can derive from extensive T-cell exhaustion and genetic aberrancies in T-cell populations [92]. These examples are not exhaustive, as there exists innumerable possible mechanisms through which tumors could evade or resist immune responses.

While clinical trials around CPI monotherapy and the combination of anti-CTLA-4 with anti-PD-1/PD-L1 did not result in high frequencies of clinical response, they help to guide subsequent clinical trials toward a more optimal treatment regimen by highlighting broad mechanisms of resistance where CPI therapies could benefit from additional or alternative agents. Clinical trials investigating these therapies and their impact on the immune system are discussed below.

### Chemotherapy

While often thought to be generally immunosuppressive, chemotherapy can be immunomodulatory with varying effects that are dependent on the type, dose, and mechanism of action of the chemotherapeutic agent employed [93]. In general, chemotherapy is utilized for several important functions: decreasing overall tumor burden, activating antigen cascades, and reducing immunosuppressive cell populations such as MDSCs and regulatory T cells [94, 95]. Since MDSCs and regulatory T cells are known to play a major role in maintaining prostate cancer immune evasion, the use of chemotherapy could bypass the mechanisms of resistance dependent on these cell populations, effectively lowering the threshold for generating an adequate antitumor immune response [27, 31].

Preclinical studies have demonstrated that this is an effective strategy for bolstering antitumor immune responses in general, and in the context of prostate cancer. In one study, the impact of treatment with paclitaxel was evaluated in a murine prostate cancer model. In these mice, the addition of paclitaxel led to a shift in macrophage populations toward the M1-like phenotype, as well as decrease in regulatory T cells [96]. Similarly, a different study in which a murine prostate cancer model received docetaxel found that treated mice had an enhanced cytotoxic T-cell response and simultaneous decreased frequency of MDSCs [97].

Additional data from case reports have also shown that oral low-dose metronomic cyclophosphamide can be utilized in order to decrease the proportion of immunosuppressive regulatory T cells, both in the TME and systemically. One patient experienced a PSA reduction of over 90% despite an aggressive tumor phenotype and poor prognosis [98]. A phase II clinical trial that followed this finding demonstrated that similar results were found in additional murine models, and that oral low-dose continuous cyclophosphamide was safe to use in patients with castration-resistant prostate cancer [99].

These results have been further refined by more specific studies in a novel murine prostate cancer model. This model did not respond well to treatment with either anti-CTLA-4 or anti-PD-1 therapy, and only modestly well to the combination of therapies, nor to MDSC-specific multikinase inhibitors (cabozantinib and BEZ235). The therapeutic benefit was thought to be derived from increased expression of IL-1R antagonist alongside decreased expression of MDSC recruiting and stimulating molecules from tumor cells [100]. At the same time, an analogous study was performed in which mice received anti-CD25 in conjunction with anti-CTLA-4 therapy. The combination of therapies led to depletion of the regulatory T-cell population and enhanced antitumor immunogenicity, significantly delaying tumor growth as compared to either treatment alone [101].

While there is evidence that chemotherapy and CPI therapy have the potential to act synergistically, there has also been recent data demonstrating the potential for chemotherapy to impact the TME in a pro-tumorigenic manner and oppose CPI therapy. This increased resistance to therapies is the result of the induction of phenotypic changes in CAFs that result in enhanced tumor chemoresistance and castration resistance. There are several mechanisms by which this occurs, including through upregulation of WNT16B in the TME leading to Wnt signaling in tumor cells, inhibition of drug accumulation at the tumor site, and prevention of chemotherapy-induced oxidative stress [102–105]. It is not yet known if this phenotypic shift in CAFs also has an impact on resistance to immunotherapies, thus representing another potential mechanism of tumor resistance to immunotherapies.

Ultimately, approved chemotherapies for treatment of mCRPC can be used safely with CPI therapy to induce an enhanced antitumor immune response. The benefit seems to be largely derived from depletion of regulatory T cells and MDSCs, reducing immunosuppression, as well as production of more and new tumor-related antigens, enhancing tumor immunogenicity.

### Radiation

Radiation naturally creates an inflammatory, immunogenic environment through direct cell DNA damage, as well as the induction of both inflammatory cytokine secretion and dendritic cell recruitment. Dendritic cells can subsequently aid in the antitumor immune response through activation of tumor-specific T cells [106]. The immunomodulatory effects of radiation are variable and derive from the dose, the degree of fractionation, and potentially the site of metastasis [107–110].

Therapeutic strategies for employing a combination of radiation and CPIs include augmentation of the induced immune response specifically at the area of disease targeted by radiation, as well as systemically. This consequence of this combination of radiation and CPI therapy is referred to as the abscopal effect [70, 71]. Radiation therapy has been shown to act synergistically with CPI therapy in patients with mCRPC. In a phase I/II clinical trial of ipilimumab and radiation, patients were enrolled regardless of the presence or absence of metastatic disease and treated with ipilimumab with or without radiation therapy. Responders were defined as those that experienced a PSA decline ≥50% at any time during the study. The combination of ipilimumab with radiation resulted in clinical responses; in both treatment arms there was a total of eight responders. While larger studies need to be undertaken to investigate the benefit of radiation with CPI therapy, the benefit was seemingly larger in the ipilimumab only subgroup: there were four responders that received radiation and ipilimumab (12%) and four responders that only received ipilimumab (25%). The rate of adverse events in regard to frequency and severity was similar to that seen in clinical trials of ipilimumab alone. Thus, there is evidence that patients with mCRPC experience an increased benefit with the addition of radiation to CPI monotherapy without any notable increase in adverse events [111].

However, a recent meta-analysis showed evidence that, while the combination of radiation and CPIs was safe for those being treated, it was not associated with an increase in overall survival in most patients. However, the positive safety profile has made the therapeutic combination a target of investigation in a variety of settings (metastatic castration-resistant disease, oligometastatic disease, high-risk local disease) [110].

The impacts of radiation on the immune system, while safe, may intrinsically hinder the efficacy of CPI therapies by limiting the inducible immune response. Radiation therapy was shown to lead to the activation of the STING/type I interferon pathway, which subsequently resulted in increased immunosuppressive inflammation. Other major mechanism through which radiation was shown to be immunosuppressive include via increasing CCR2-mediated recruitment of MDSCs to the tumor, as well as upregulating the production and secretion of TGF-beta [112, 113].

Overall, radiation therapy is safe and leads to immunogenic benefits in patients, but its use in conjunction with CPIs may be hindered by the resulting enhancement of an immunosuppressive TME. It can be presumed that an optimal circumstance in which to use radiation therapy would be in patients with bony metastases and with a low baseline density of MDSCs in the TME, whereas radiation may be a suboptimal therapy in those with tumors that rely more heavily on MDSCs to induce immunosuppression.

### PARP inhibitors

The utilization of PARP inhibitors alongside CPIs has been investigated in multiple settings, resulting in a number of different mechanisms through which the combination can act synergistically. PARP inhibitors have been proposed to lead to a clinical benefit via disruption of single-strand DNA breaks. This has been shown to involve cyclic GMP-AMP synthase-stimulator pathway-mediated upregulation of type I interferon signaling secondary to the presence of cytosolic DNA, as well as disruptions in interferon signaling [114]. In addition, in a study that explored the immunomodulatory effects of PARP inhibitors in a breast cancer model, exposure to PARP inhibition was associated with increased expression of PD-L1, and that use of both CPI therapy with PARP inhibitors was superior in regards to treatment efficacy as compared to either treatment alone [115]. Thus, PARP inhibitors can potentially circumvent mechanisms of resistance against CPI therapies that involve tumor sequestration of TSAs, as well as those that hinder T-cell activation and proliferation via disrupted interferon-gamma signaling.

PARP inhibitors have also been investigated in a clinical setting within the context of mCRPC. Olaparib monotherapy was shown to be superior to physician’s choice of enzalutamide or abiraterone in patients with mCRPC and confirmed alterations in one of 15 predefined DDR genes. The trial enrolled 387 patients into one of two arms: those with alterations in *BRCA1*, *BRCA2*, or *ATM* (cohort A) vs. those with alterations in one of the 12 other DDR genes but unaltered *BRCA1*, *BRCA2*, and *ATM* (cohort B). Olaparib led to a significant benefit in regard to radiographic PFS and ORR in both cohorts, with those in cohort A realizing the most substantial benefit (rPFS = 7.39 months vs. 3.55 months, HR 0.34, *p* < 0.0001). Interim overall survival was also increased, but to a nonsignificant degree, in those that were treated with olaparib despite allowed crossover from cohort B after central review (cohort A: 18.50 vs. 15.11 months, *p* = 0.0173 [alpha spend 0.01]; cohort B: 17.51 vs. 14.26 months, *p* = 0.0063 [nominal]). Thus, olaparib is superior to the anti-androgens enzalutamide and abiraterone in patients with mCRPC and alterations in DDR genes [116]. This study highlighted that PARP inhibitors can lead to a potential clinical benefit in the setting of mCRPC with dysfunctional DDR pathways, even when administered as monotherapy. This is presumably due to the combination of enhanced frequency of DNA mutations from DDR gene alterations and olaparib-mediated stimulation of the immune system, but the exact mechanism remains unknown.

The combination of olaparib with CPI therapy have led to results consistent with the discussed benefit of olaparib monotherapy for patients with mCRPC and DDR alterations. In one phase II clinical trial, olaparib was explored in combination with an anti-PD-L1 antibody, durvalumab. Of the 17 patients that were enrolled, nine were found to have evidence of response based on radiographic and/or biochemical data. This response was associated with a 12-month PFS of 51.5% (95% CI = 25.7–72.3%). However, a majority of the responders (five of nine) were found to have evidence of dysfunctional DDR genes via genomic analysis. When investigators stratified patients according to presence or absence of mutations in DDR genes, patients with mutated DDR genes were found to have a significantly higher 12-month PFS of 83.3% (*p* = 0.031; 95% CI = 27.3–94.5%) than those without evidence of mutations in DDR genes (12-month PFS = 36.4%; 95% CI = 11.2–62.7%) [117]. Although it is evident that the use of olaparib, both alone and with durvalumab, led to a durable benefit in patients with DDR gene alterations, this patient population also tends to respond better to anti-androgen and CPI therapies compared to patients without DDR gene alterations, as seen in the discussed clinical trials of nivolumab with ipilimumab [86, 87, 118]. Thus, while there is evidence that PARP inhibitors can enhance tumor immunogenicity, the mechanism by which it acts also overlaps to a degree with other genotoxic therapies, such as radiation. Results from currently ongoing clinical trials will help to determine the comparative benefit of PARP inhibitors over other genotoxic therapies, as well as to optimize the use of PARP inhibitors as monotherapy or with other agents as well as are ongoing randomized controlled trials that compare the efficacy of the combination of PARP inhibitors with CPIs in prostate cancer (e.g., NCT03834519).

### Antiangiogenics

As mentioned earlier, prostate tumors are associated with alterations in the structure and function of local vasculature, which promotes an immunosuppressive TME and enables effective evasion of the immune system. The consequences of the dysregulation of local vasculature include an increase in regulatory T cells, induction of a phenotypic shift in TAMs toward the M2-like immunosuppressive phenotype, decreased dendritic cell maturation and antigen presentation, and increased endothelial cell PD-L1 expression [5]. Conversely, in preclinical models, improved regulation of local vasculature was shown to [1] promote assimilation of TAMs with the M1-like phenotype [2], increase CD4+ and CD8+ T-cell infiltration into the TME, and [3] decrease the overall MDSC population [119]. Thus, antiangiogenic therapy promotes an immunogenic TME through inhibition of tumor-induced dysregulation of local vasculature and the hypoxia-associated increases in immunosuppressive cell populations.

Multiple studies have been conducted to explore the immunomodulatory effects of antiangiogenics and the potential synergistic mechanisms of action they have when used in conjunction with CPIs. In a phase Ib clinical trial, investigators explored the efficacy of the combination of cabozantinib, a multiple receptor tyrosine kinase inhibitor, with atezolizumab in patients with mCRPC that had been previously treated with abiraterone and/or enzalutamide and subsequently shown to have disease progression. In 44 patients with RECIST-measurable disease and an ECOG status of 0 or 1, it was found that treatment with the combination of cabozantinib and atezolizumab resulted in an objective response rate of 32% (14 patients; 80% CI = 23–42), with two patients showing complete response and 12 patients showing a partial response. Of the 12 responders that had at least one post-baseline PSA evaluation, PSA declined ≥50% in 8 of them. In addition, it was found that 21 patients had stable disease, and thus treatment with this combination resulted in a disease control rate of 80% [120].

These results, when interpreted in the context of the aforementioned preclinical data, highlight the synergism present between PARP inhibitors and CPI therapies. Since PARP inhibitors attenuate the immunosuppressive functions of TAMs and MDSCs while increasing the ratio of antitumor TILs to regulatory T cells, they can contribute to overcoming the mechanisms of resistance to CPI therapies that involve or are dependent on M2-like TAMs, MDSCs, and a low ratio of antitumor TILS to regulatory T cells. Moreover, resistance to anti-PD-L1 is in part due to exclusion of antitumor CD8+ T cells from the TME, and resistance to enzalutamide is associated with high expression of PD-L1 [84, 121]. PARP inhibitors are thus an optimal therapy to be used in combination with anti-PD-L1 CPI therapy, and the two therapies together target main mechanisms of persistent immune evasion employed by enzalutamide-resistant prostate cancer.

However, results of the phase Ib trial were not replicated in larger clinical trials of PARP inhibitors. In one phase III trial, chemotherapy-naïve patients with progressive mCRPC were randomized to receive docetaxel with prednisone and either bevacizumab or placebo. While there was a significant increase in PFS and ORR, there was no statistical difference in median overall survival between the two groups (22.6 vs. 21.5 months; HR = 0.91, 95% CI = 0.78–1.05). There was also a statistically significant increase in grade 3 or greater treatment-related toxicities, as well as treatment-related deaths in patients that received bevacizumab [122]. Therefore, patients that received docetaxel with prednisone and bevacizumab displayed evidence of enhanced antitumor immune activity, but did not lead to sufficient tumor cell death to manifest a durable response.

The lack of durable response with docetaxel and bevacizumab in the setting of chemotherapy-naïve mCRPC despite evidence of antitumor activity can be interpreted as an initial response to therapy and subsequent development of resistance, or as intrinsic tumor resistance to both therapies. Larger clinical trials, as well as more granular data describing the prior treatments received, are needed to further highlight the mechanisms that are behind resistance to chemotherapy in conjunction with PARP inhibitors.

### T-cell engagers

The immunotherapies discussed thus far rely on cancer patients possessing endogenous T cells that can recognize their cancer. For patients that lack these T cells, alternative strategies are needed. Bispecific monoclonal antibodies (BSAbs) are designed to generate antitumor immune responses by crosslinking tumor-specific cell surface antigens with the coreceptors on T cells. While efficacy of BSAbs in the setting of prostate cancer is currently under investigation, preclinical data have suggested promising therapeutic impacts. Buhler et al. originally published one of the first studies to conduct successful experiments involving the use of BSAbs to target prostate cancer. In this study, tetramers that targeted both PSMA, a TSA, and CD3 (CD3xTSA) were synthesized and then tested in vitro using a murine model of severe combined immunodeficiency inoculated with prostate cancer tumor cells. These mice received treatment with peripheral blood lymphocytes alone or in combination with the experimental CD3xTSA BSAb. Analysis highlighted a significant difference in tumor size between the two treatment arms, marking the potential benefit that could be realized from CD3xTSA BSAb-based immunotherapies [123]. More recently, a study that also utilized murine xenograft tumor models demonstrated similar results. Mice that received treatment with a CD3xTSA BSAb instead of an empty treatment vehicle experienced significantly higher rates of tumor regression and overall improved survival [124].

A preclinical study revealed that AMG 160, a similar CD3xTSA BSAb, resulted in the upregulation of PD-1 expression in T cells when assessed in vitro using human prostate cancer explant models. Combination therapy utilizing AMG 160 with anti-PD-1 therapy led to enhanced cell killing when compared to the use of AMG 160 alone. Specifically, researchers found that AMG 160 alone led to the activation of TILs in the TME, and that this effect was augmented when combined with anti-PD-1 therapy [125].

In addition, there are numerous clinical trials being carried out to assess the safety and efficacy of BSAbs as treatment for prostate cancer. In one such trial, patients with mCRPC were treated with pasotuxizumab, a CD3xTSA BSAb targeting the prostate-specific membrane antigen (PMSA). The results of this trial showed that, of the 16 patients that received treatment with pasotuxizumab, there was a ≥50% decline in PSA for three patients, with decreases in PSA occurring in a dose-dependent manner. Two of the three patients with PSA declines experienced prolonged response (14 months, 19.4 months), with one of the patients experiencing near complete regression of metastases alongside a >90% decline in PSA and alkaline phosphatase [126].

While the majority of BSAbs trialed in the setting of prostate cancer thus far have utilized CD3 in order to target T cells, recent studies have shown that targeting CD28 and a TSA (CD28xTSA) can act synergistically with CD3xTSA BSAbs. Used independently, CD28xTSA BSAbs led to limited activity and minimal toxicity. However, the coadministration of CD28xTSA BSAbs with CD3xTSA significantly potentiated the ability of CD3-targeted BSAbs to induce an antitumor immune response, with data reflecting a 50-fold shift in the median effective concentration. Simultaneously, use of both agents led to a significantly increased maximal induction of interferon-gamma release, while expanding and activating CD4+ and CD8+ T cells [127]. Subsequently the efficacy of the PMSA-targeting CD28xTSA BSAb, named REGN5678, was investigated when used alongside PD-1 blockade. In a murine prostate cancer model, this combination of therapies improved antitumor immune responses. These studies have led to the initiation of a phase I/II clinical trial assessing the safety and efficacy of REGN5678 in combination with the anti-PD-1 CPI therapy cemiplimab, and results are still pending.

### Adenosine pathway inhibitors

The adenosine signaling pathway has recently been recognized as a targetable component of cancer immune evasion and is of relevance to the treatment of prostate cancer given that PAP generates adenosine [128, 129]. Pharmacologically, the adenosine signaling pathway can be disrupted by targeting one of several of critical steps. Signal transduction can be inhibited via blockade of A2AR or adenosine B2 receptor, and similarly, key upstream reactions can be inhibited via blockade of nucleotidases that are involved in metabolism of nicotinamide adenine dinucleotide or adenosine diphosphate, such as CD73 [53]. The immunomodulatory effects of the adenosine signaling pathway and its therapeutic blockade are especially important in the setting of prostate cancer, as PAP acts as an ectonucleotidase that locally generates adenosine [128, 129].

Recently, Sidders et al. discovered and validated a gene expression signature that can be used to identify patients with prostate tumors that utilize adenosine signaling pathways in order to prevent an adequate antitumor immune response. After applying the gene expression signature to patients from The Cancer Genome Atlas and cohorts of patients that had been treated with CPIs. The signature was shown to significantly correlate with baseline adenosine levels (*r*^2^ = 0.92; *p* = 0.018), and was absent in A2AR-knockout mice. In addition, the gene expression signature decreased during corresponding treatment with A2AR inhibitors in mouse models (*r*^2^ = −0.62; *p* = 0.001) and five of seven humans. Their analysis of patients from The Cancer Genome Atlas further evidenced the negative prognostic capacity of adenosine, as it was significantly associated with decreased overall survival (HR = 0.6; *p* < 2.2*e*^−16^) and decreased PFS (HR = 0.77, *p* = 6*e*^−7^). In the presence of CD+ TILs, adenosine signaling was also significantly associated with decreased overall survival (HR = 0.47; *p* < 2.2*e*^–16^) and PFS (HR = 0.65, *p* = 2*e*^−7^). Last, adenosine signaling was significantly associated with increased resistance to anti-PD-1 CPI in published cohorts of patients, highlighting the clinical importance of identifying patients in which adenosine signaling contributes to reduced treatment efficacy [130]. The results of this study emphasize a potential method through which clinicians can better predict response to CPIs and therapies that target the adenosine signaling pathway, while highlighting the magnitude of the role that adenosine can have in promoting resistance to therapeutic interventions.

Another recent preclinical trial of CPI-444, an A2AR inhibitor, further bolstered the growing body of evidence around the benefits of adenosine blockade, especially when used in conjunction with CPIs. Mice were exposed to a viral challenge and then treated with CPI-444 3 days later in order to evaluate the impact of A2AR inhibition on CD8+ T cells. Treatment with CPI-444 was associated with an enhanced antigen-specific CD8+ T-cell response, as well as decreased expression of several immune checkpoints in CD8+ T cells, including PD-1, TIM-3, and LAG-3. A similar decrease in immune checkpoints was also noted in regulatory T cells alongside a decrease in the expression of FoxP3. Further experiments in two murine models of colon cancer reflected a reduction in tumor growth that was significant in one of the models and a similar nonsignificant trend in the other model. In addition, CPI-444 was associated with enhanced tumor regression and animal survival when combined with anti-PD-1 therapy in both of the aforementioned murine models. Of note, data showed that the most potent effect of adenosine signaling pathway inhibition occurred at tumor draining lymph nodes, in which CD8 + CD44+ T cells had significantly reduced expression of PD-1 and LAG-3 in contrast to control mice treated with placebo [131]. If similar findings hold true in humans, this evidence suggests that adenosine signaling pathway blockade would be most effective shortly after exposure of T cells to a new antigen and could enhance the efficacy of anti-PD-1 antibody therapy when used in conjunction with one another. Targeting adenosine thus represents a strategy that can be used to bypass resistance to CPI therapy induced by the expression of inhibitory checkpoint molecules aside from CTLA-4 and PD-L1.

In addition to preclinical trials, several clinical trials have taken place to evaluate the efficacy of therapies that target the adenosine signaling pathway. A phase I clinical trial of AZD4653, an A2AR inhibitor, with or without simultaneous treatment with durvalumab was conducted in patients with mCRPC and RECIST-measurable disease. The results of this trial demonstrated that there was an overall confirmed response rate of 37.5% throughout the cohort. In one of the four patients evaluated, there was a durable decline in PSA by >99% [132]. In a separate, ongoing phase I clinical trial, patients with RECIST-measurable mCRPC that had previously received up to five systemic therapies were enrolled to assess the efficacy and safety of ciforadenant, a potent A2AR inhibitor, alone or in combination with atezolizumab. Of the reported 14 evaluable patients that were enrolled, five experienced tumor regression and eight were found to have stable disease, leading to a clinical benefit rate of 8/14 (57%). In addition, the median duration of disease control was found to be 24 months [133].

Combination A2AR and A2BR antagonist therapy for the treatment of mCRPC is being evaluated in a phase Ib/II clinical trial of AB928, a small molecule dual antagonist. Cohorts of patients with mCRPC will be treated with either (1) AB928 and AB122, an anti-PD-1 antibody, in conjunction with standard of care (enzalutamide or docetaxel), or (2) AB928 in combination with AB680, a CD73 inhibitor, with or without AB122 [134]. Results of this trial and other ongoing trials are critical for understanding the specific mechanism through which adenosine antagonism enhances immunogenicity in prostate cancer.

## Future directions

Prostate cancer has the potential to be immunogenic but generates an immunosuppressive TME capable of resisting CPI therapies through recruitment of inhibitory immune cell populations, production of pro-tumorigenic cytokines, and direct inhibition of the immune system by tumor cells. Therefore, unlike other cancers where blocking the PD-1/PD-L1 axis alone might be sufficient, prostate cancer will require a combination of approaches to improve the rates of clinical responses. The results of currently ongoing phase II and III clinical trials will provide insights into the advantages and disadvantages to different strategies, as well as the mechanisms of therapeutic resistance that have yet to be overcome (NCT03753243, NCT03810105, NCT03879122, NCT03016312, NCT03338790).

Moreover, as will be discussed in the accompanying review, patient selection may also need to be incorporated into defining the prostate cancer patients that will be responsive to specific treatments. While certain patient populations have been identified that respond advantageously to immunotherapy, such as those with microsatellite instability, dysfunctional DDR, or *CDK12* alterations, these populations do not encompass the majority of patients [135–137]. While many of the aforementioned clinical trials will also provide evidence for optimizing patient selection based on tumor-intrinsic molecular features, identification of clinically applicable immune signatures may represent another approach to improve patient selection. With emerging use of high-dimensional single-cell analyses to assess tumors, we may not only elucidate other mechanisms of resistance, but also potentially develop more robust biomarkers to help guide the selection of immunotherapies for prostate cancer.
